# Immunofluorescence Analysis of NF-kB and iNOS Expression in Different Cell Populations during Early and Advanced Knee Osteoarthritis

**DOI:** 10.3390/ijms22126461

**Published:** 2021-06-16

**Authors:** Marko Ostojic, Ante Zevrnja, Katarina Vukojevic, Violeta Soljic

**Affiliations:** 1 Department of Orthopaedics and Traumatology, University Hospital Mostar, 88 000 Mostar, Bosnia and Herzegovina; marko.ostojic@mef.sum.ba; 2 Department of Anatomy, School of Medicine, University of Mostar, 88 000 Mostar, Bosnia and Herzegovina; 3 Public Health Centre, First Responders Unit, 21 000 Split, Croatia; antezevrnja17@gmail.com; 4 Department of Anatomy, Histology and Embryology, School of Medicine, University of Split, Soltanska 2, 21 000 Split, Croatia; 5 Department of Histology and Embryology, School of Medicine, University of Mostar, Kralja Petra Kresimira IV, 88 000 Mostar, Bosnia and Herzegovina; violeta.soljic@mef.sum.ba

**Keywords:** osteoarthritis, synovitis, inducible nitric oxide synthase, nuclear factor kappa B, macrophage

## Abstract

Synovitis of the knee synovium is proven to be a precursor of knee osteoarthritis (OA), leading to a radiologically advanced stage of the disease. This study was conducted to elucidate the expression pattern of different inflammatory factors—NF-kB, iNOS, and MMP-9 in a subpopulation of synovial cells. Thirty synovial membrane intra-operative biopsies of patients (ten controls, ten with early OA, and ten with advanced OA, according to the Kellgren–Lawrence radiological score) were immunohistochemically stained for NF-kB, iNOS, and MMP9, and for different cell markers for macrophages, fibroblasts, leukocytes, lymphocytes, blood vessel endothelial cells, and blood vessel smooth muscle cells. The total number of CD68+/NF-kB+ cells/mm^2^ in the intima of early OA patients (median = 2359) was significantly higher compared to the total number of vimentin+/Nf-kB+ cells/mm^2^ (median = 1321) and LCA+/NF-kB+ cells/mm^2^ (median = 64) (*p* < 0.001 and *p* < 0.0001, respectively). The total number of LCA+/NF-kB+ cells/mm^2^ in the subintima of advanced OA patients (median = 2123) was significantly higher compared to the total number of vimentin+/NF-kB+ cells/mm^2^ (median = 14) and CD68+/NF-kB+ cells/mm^2^ (median = 29) (*p* < 0.0001). The total number of CD68+/iNOS+ cells/mm^2^ in the intima of both early and advanced OA patients was significantly higher compared to the total number of vimentin+/iNOS+ cells/mm^2^ and LCA+/iNOS+ cells/mm^2^ (*p* < 0.0001 and *p* < 0.001, respectively). The total number of CD68+/MMP-9+ cells/mm^2^ in the intima of both early and advanced OA patients was significantly higher compared to the total number of vimentin+/MMP-9+ cells/mm^2^ and CD5+/MMP-9+ cells/mm^2^ (*p* < 0.0001). Macrophages may have a leading role in OA progression through the NF-kB production of inflammatory factors (iNOS and MMP-9) in the intima, except in advanced OA, where leukocytes could have a dominant role through NF-kB production in subintima. The blocking of macrophageal and leukocyte NF-kB expression is a possible therapeutic target as a disease modifying drug.

## 1. Introduction

Osteoarthritis (OA) is the most common disease of the synovial joints that mostly affects older adults. It indicates chronic degenerative changes in the articular cartilage that perpetuate pathological changes in other parts of the joint in a cascade manner [1]. Degenerative changes in the cartilage are both in biochemical composition and physical properties [2,3]. The pronounced pathological changes of knee osteoarthritis include cartilage damage, osteophyte formation, subchondral bone sclerosis, synovial inflammation, the degeneration of ligaments and the menisci, and the thickening of the joint capsule [1]. Current management largely emphasizes on alleviating symptoms and improving function, but for many patients, these measures do not provide adequate symptom relief [4]. With the detection of high levels of cytokines, complement and plasma proteins in the synovial fluid, it has been acknowledged that OA is a low-grade inflammatory disease with underlying mechanical disorders accelerating the failure of the joint [5,6,7]. Therefore, investigations of different cell populations within the synovial membrane and the expression patterns of inflammatory markers can elucidate the relationship between diagnosis and prognostic outcome [8]. The synovial membrane contains resident cells such as macrophage-like synoviocytes (MLS), fibroblast-like synoviocytes (FLS), endothelium and smooth muscle cells of blood vessels, and non-resident, inflammatory cells, such as lymphocytes and plasma-cells. It has two layers; the intima, the thin layer where the macrophages and fibroblasts are located, and the subintima, the supportive stroma [9]. MLS in the synovial membrane of OA joints contribute to cartilage breakdown by producing inflammatory cytokines as well as matrix metalloproteinases (MMPs) that lead to synovitis development [5,8]. Synovial inflammation, together with subchondral bone marrow edema, has more significance in the early phases of the disease as a result of initial cartilage lesions [10,11,12]. In early OA, the synovial membrane shows synovial lining cell thickness, vascularity and the expression of inflammatory cells and mediators, and nuclear transcription factors to be greater in advanced OA, determining a higher hyperplastic and inflammation component of the synovitis in that stage [11,13]. Additionally, clinically, synovitis grade is greatly associated with the severity of pain, joint dysfunction, and cartilage loss in OA [14,15].

After the initial cartilage damage, parts of the cartilage cells and matrixes are released into the synovial fluid [5,16]. Cells in the cartilage and in the synovial membrane produce inflammatory mediators, such as cytokines, angiogenic factors, chemokines, MMPs, COX-2, and inducible nitric oxide synthase (iNOS), that play a distinctive role in the further degradation of the cartilage [8,17]. These inflammatory factors are mostly produced through NF-kB transcription factor activity by the MLS in the synovial intima [8,18]. In OA, NF-kB is highly activated at sites of synovial membrane inflammation, inducing transcription of the aforementioned proinflammatory factors [19]. iNOS is located in the cartilage and synovial membrane and is mostly synthetized through the NF-kB pathway [20]. Nitric oxide (NO) is produced by iNOS, and it mediates the expression of inflammatory factors, inhibits the synthesis of collagen and proteoglycans, and induces chondrocyte apoptosis and pain [21]. Selective inhibition of iNOS reduces the tissue levels of catabolic factors; therefore, NO has an inflammatory role in OA [22]. On the contrary, some studies show a possible protective role of NO through the NF-kB feedback mechanism [23]. Different MMPs have been detected in the synovial fluid of patients with OA and have been directly correlated with the expression of inflammatory cells in the synovial membrane [24,25,26]. 

FLS and MLS are the most active innate immunity cells in inflamed synovial tissue in early osteoarthritis [27,28,29,30,31,32,33]. So far, only a few studies have tried to determine the differences between NF-kB, iNOS, and MMPs expression in the different cells of knee synovial tissues in early and advanced OA, and all of them were focused on MLS and FLS [27,31,32], while studies investigating the role of cells within the blood vessels and lymph nodules of the knee synovial membrane in OA are scarce [34]. 

Newer treatment aspirations aim for early diagnosis and the usage of disease modifying drugs that could halt the progression of OA [35,36]. There is a theory that one subpopulation of OA patients was “synovium driven” and that specifically aiming the synovitis can be a successful treatment modality [8]. Therefore, the aim of this study was to analyze the expression pattern of NF-kB, iNOS, and MMP-9 in different cells that constitute the synovial membrane in the early and advanced stages of OA according to the radiological stage of OA. Most other studies did not have a control group, and to the best of our knowledge, neither one differentiated between the two main layers of the synovial membrane. Understanding the immunophenotype of the different cells of the knee synovial tissues can help us in the development of a potential therapeutical target that can influence disease progression.

## 2. Results

**Figure 1:**
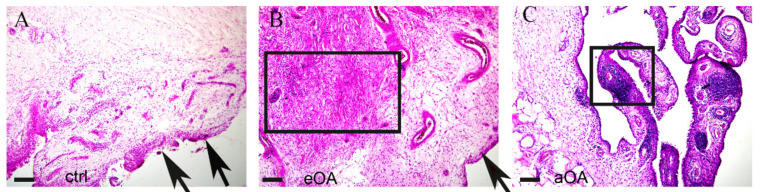
Hystological staining of healthy (ctrl) (**A**), early osteoarthritic synovium (eOA) (**B**), and advanced osteoarthritic synovium (aOA) (**C**). The synovial membrane of patients in the control group shows no resident cells in the subintima and the intima (arrows). In patients with early OA, the synovium contains highly visible cell infiltration (frame) in the intima (arrow). In patients with advanced OA, lymphoid nodules (frame) are shown. Haematoxylin-eosin staining. Magnification ×20, scale bar 50 µm.

Biopsies of the synovial membranes were divided into early OA, advanced OA, and control groups (Figure 1) according to the radiologically based Kellgren–Lawrence scale (Table 1).

**Figure 2:**
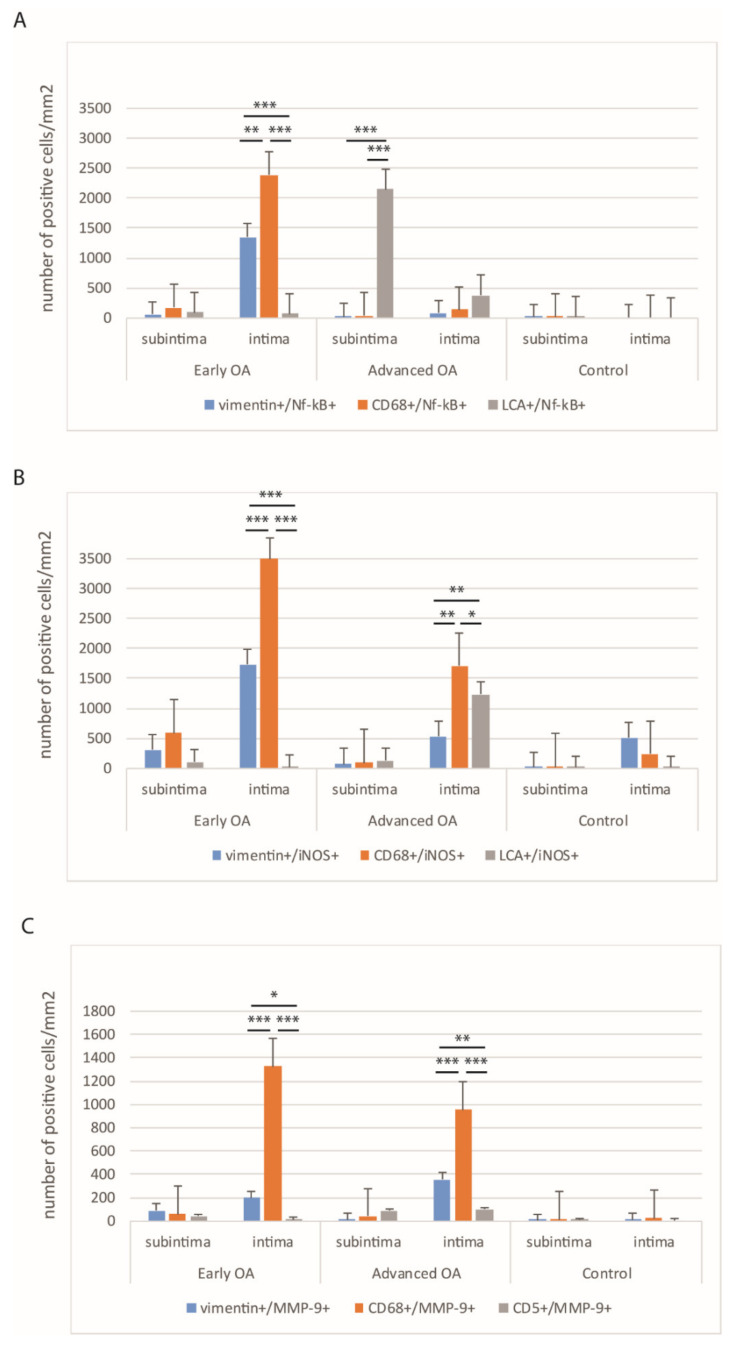
The distribution of vimentin+/NF-kB+, CD68+/NF-kB+, and LCA+/NF-kB+ (**A**), vimentin+/iNOS+, CD68+/iNOS+, and LCA+/iNOS+ (**B**), and vimentin+/MMP-9+, CD68+/MMP-9+, and CD5+/MMP-9 (**C**) positive cells per mm^2^ in the early osteoarthritis (OA), in the advanced osteoarthritis (OA) and control groups. Data were shown as mean ± SD. Significant differences (Kruskal–Wallis) are indicated by * *p* < 0.05, ** *p* < 0.001, *** *p* < 0.0001.

**Figure 3:**
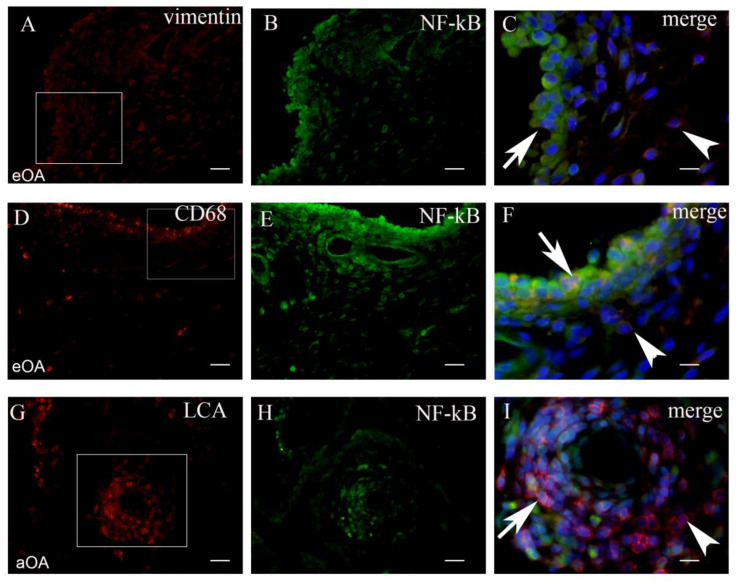
The synovial membrane of patients with early OA (eOA) (**A**–**C**). Vimentin positive (red) fibroblasts in the intima and subintima (frame), NF-kB positive cells (green) in the synovial intima and subintima (**B**). The merge of DAPI (blue) nuclear stain+vimentin+NF-kB (magnification of the frame in panel (**A**)) show numerous fibroblasts in the intima co-localized with NF-kB and vimentin (arrow), while the subintima displays vimentin positive fibroblasts (arrowhead) (**C**). The synovial membrane of patients with early OA (eOA) (**D**–**F**). CD68 positive (red) macrophages in the intima and subintima (frame). NF-kB positive cells (green) in the synovial intima and subintima (**E**). The merge of DAPI (blue)+CD68+NF-kB (magnification of the frame in panel (**D**)) show numerous macrophages in the intima co-localized with NF-kB and CD68 (arrow), while the subintima displayed CD68 positive macrophages(arrowhead) (**F**). The synovial membrane of patients with advanced OA (aOA) (**G**–**I**). LCA positive (red) lymphocytes in the intima, subintima, and lymph nodule (frame). NF-kB positive cells (green) in the synovial intima and subintima (**H**). The merge of DAPI (blue)+LCA+NF-kB (magnification of the frame in panel (**G**)) show numerous lymphocytes in the lymph nodule co-localized with NF-kB and LCA (arrow), while some lymphocytes displayed only LCA (arrowhead) (**I**). Magnification ×40 (first two columns), scale bar 25 µm and ×100 (last column), scale bar 10 µm.

**Figure 4:**
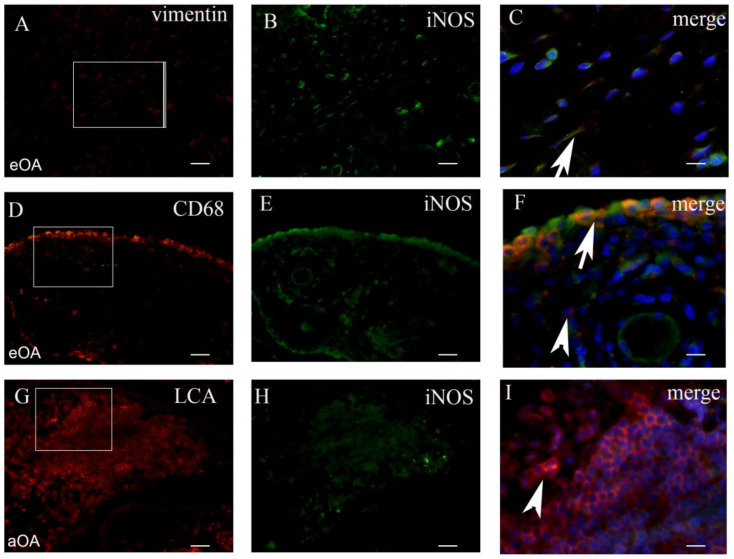
The synovial membrane of patients with early OA (eOA) (**A**–**C**). Vimentin positive (red) fibroblasts in the subintima (frame). iNOS positive cells (green) in the synovial intima and subintima (**B**). The merge of DAPI (blue) nuclear stain+vimentin+iNOS (magnification of frame in panel **A**) show occasional fibroblasts in the subintima co-localized with iNOS and vimentin (arrow) (**C**). The synovial membrane of patients with early OA (eOA) (**D**–**F**). CD68 positive (red) macrophages in the intima and subintima (frame). iNOS positive cells (green) in the synovial intima and subintima (**E**). The merge of DAPI (blue)+CD68+iNOS (magnification of the frame in panel (**D**)) show numerous macrophages in the intima co-localized with iNOS and CD68 (arrow), while the subintima displayed CD68 positive macrophages(arrowhead) (**F**). The synovial membrane of patients with advanced OA (aOA) (**G**–**I**). LCA positive (red) lymphocytes in the intima, subintima, and lymph nodule (frame). iNOS positive cells (green) in the synovial intima and subintima (**H**). The merge of DAPI (blue)+LCA+iNOS (magnification of the frame in panel (**G**)) show no co-localization of iNOS and LCA, while a numerous number of lymphocytes display only LCA (arrowhead) (**I**). Magnification ×40 (first two columns), scale bar 25 µm and ×100 (last column), scale bar 10 µm.

**Figure 5:**
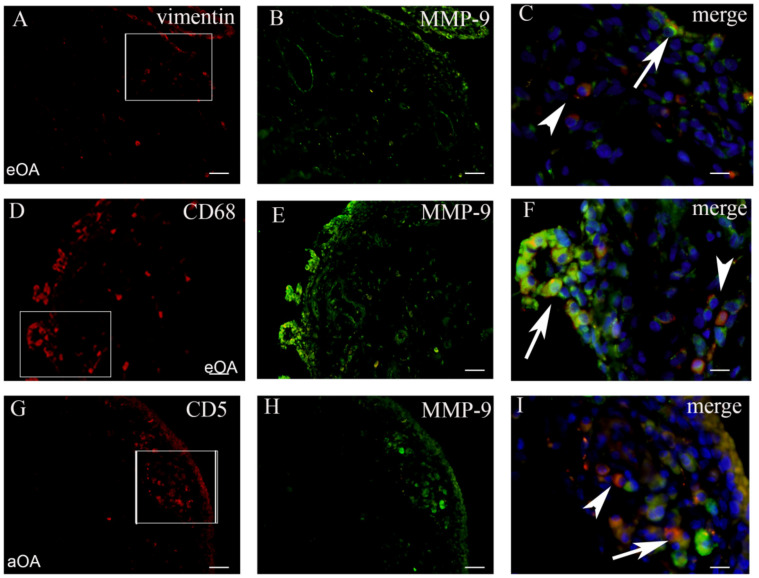
The synovial membrane of patients with early OA (eOA) (**A**–**C**). Vimentin positive (red) fibroblasts in the intima and subintima (frame). MMP-9 positive cells (green) in the synovial intima and subintima (**B**). The merge of DAPI (blue) nuclear stain+vimentin+MMP-9 (magnification of the frame in panel (**A**)) show numerous fibroblasts in the intima co-localized with MMP-9 and vimentin (arrow), while the subintima displayed vimentin positive fibroblasts (arrowhead) (**C**). The synovial membrane of patients with early OA (eOA) (**D**–**F**). CD68 positive (red) macrophages in the intima and subintima (frame). MMP-9 positive cells (green) in the synovial intima and subintima (**E**). The merge of DAPI. (blue)+CD68+MMP-9(magnification of the frame in panel (**D**)) show numerous macrophages in the intima co-localized with MMP-9 and CD68 (arrow), while the subintima displayed CD68 positive macrophages(arrowhead) (**F**). The synovial membrane of patients with advanced OA (aOA) (**G**–**I**). CD5 positive (red) lymphocytes in the subintima (frame). MMP-9 positive cells (green) in the synovial intima and subintima (**H**). The merge of DAPI (blue)+CD5+iNOS (magnification of the frame in panel (**G**)) show the co-localization of MMP-9 and CD5 (arrow), while lymphocytes occasionally display only CD5 (arrowhead) (**I**). Magnification ×40 (first two columns), scale bar 25 µm and ×100 (last column), scale bar 10 µm.

Tissue sections were analyzed with the double immunofluorescence of NF-kB, iNOS, and MMP-9, with prospective markers for different cell populations of the synovial intima and subintima (Figure 2, Figure 3, Figure 4 and Figure 5). Namely, CD31 and actin were used as markers for the blood vessel wall. Vimentin was used as a marker for fibroblasts, LCA served as a marker for leukocytes while CD68 and CD5 were used as markers for macrophages and T lymphocytes, respectively.

### 2.1. Co-Localization of NF-kB+ Cells and Markers of Cell Populations in Synovial Intima and Subintima

The total number CD68+/NF-kB+ cells/mm^2^ in the intima of early OA (median = 2359) was significantly higher compared to the total number of vimentin+/NF-kB+ cells/mm^2^ (median = 1321) and LCA+/NF-kB+ cells/mm^2^ (median = 64) (*p* < 0.001 and *p* < 0.0001, respectively). The total number of vimentin+/NF-kB+ cells/mm^2^ in the intima of early OA patients was significantly higher compared to the total number LCA+NF-kB+ cells/mm^2^ (*p* < 0.0001). The difference between CD68+/NF-kB+ (median = 172), vimentin+/NF-kB+ (median = 53), and LCA+/NF-kB+ cells/mm^2^ (median = 85) in the subintima of early OA was not statistically significant. 

The total number of LCA+/NF-kB+ cells/mm^2^ in the subintima of advanced OA patients (median = 2123) was significantly higher compared to the total number of vimentin+/NF-kB+ cells/mm^2^ (median = 14) and CD68+/NF-kB+ cells/mm^2^ (median = 29) (*p* < 0.0001; and *p* < 0.0001, respectively). The difference between LCA+/NF-kB+ (median = 368), vimentin+/NF-kB+ (median = 74), and CD68+/NF-kB+ cells/mm^2^ (median = 131) in the intima of advanced OA patients was not statistically significant. These results were graphically summarized in Figure 2A, and positive cells were shown in Figure 3.

### 2.2. Co-Localization of iNOS+ Cells and Markers of Cell Populations in Synovial Intima and Subintima

The total number of CD68+/iNOS+ cells/mm^2^ in the intima of early OA patients (median = 3492) was significantly higher compared to the total number vimentin+/iNOS+ cells/mm^2^ (median = 1729) and LCA+/iNOS+ cells/mm^2^ (median = 25) (*p* < 0.0001; and *p* < 0.0001, respectively). The total number of vimentin+/iNOS+ cells/mm^2^ in the intima of early OA patients was significantly higher compared to the total number of LCA+/iNOS+ cells/mm^2^ (*p* < 0.0001). The difference between CD68+/iNOS+ (median = 589), vimentin+/iNOS+ (median = 301), and LCA+/iNOS+ cells/mm^2^ (median = 102) in the subintima of early OA patients was not statistically significant. 

The total number of CD68+/iNOS+ cells/mm2 in the intima of advanced OA (median = 1701) was significantly higher compared to the total number of vimentin+/iNOS+ cells/mm^2^ (median = 532) and LCA+/iNOS+ cells/mm^2^ (median = 1233) (*p* < 0.01; and *p* < 0.001, respectively). The total number of LCA+/iNOS+ cells/mm^2^ in the intima of advanced OA patients was significantly higher compared to the total number of vimentin+/iNOS+ cells/mm^2^ (*p* < 0.001). The difference between CD68+/iNOS+ (median = 91), vimentin+/iNOS+ (median = 68), and LCA+/iNOS+ cells/mm^2^ (median = 128) in the subintima of advanced OA was not statistically significant. These results were graphically summarized in Figure 2B, and positive cells were shown in Figure 4.

### 2.3. Co-Localization of MMP+ Cells and Markers of Cell Populations in Synovial Intima and Subintima

In order to confirm the expression of MMP-9 in different synovial membrane cells, we analyzed for the co-localization of the MMP-9 marker and prospective markers for fibroblasts, macrophages, and lymphocytes (Figure 5).

In early OA patients, macrophages strongly expressed MMP-9 (Figure 5). The total number of CD68+/MMP-9+ cells/mm^2^ in the intima of early OA patients (median = 1320) was significantly higher compared to the total number of vimentin+/MMP-9+ cells/mm^2^ (median = 205) and CD5+/MMP-9+ cells/mm^2^ (median = 11) (*p* < 0.0001). The total number of vimentin+/MMP-9+ cells/mm^2^ in the intima of early OA patients was significantly higher compared to the total number of CD5+/MMP-9+ cells/mm^2^ (*p* < 0.01). Similarly, for early OA patients, the total number of CD68+/MMP-9+ cells/mm^2^ in the intima of advanced OA (median = 948) was significantly higher compared to the total number of vimentin+/MMP-9+ cells/mm^2^ (median = 361) and CD5+/MMP-9+ cells/mm^2^ (median = 93) (*p* < 0.0001). The total number of vimentin+/MMP-9+ cells/mm2 in the intima of advanced OA patients was significantly higher compared to the total number of CD5+/MMP-9+ cells/mm^2^ (*p* < 0.001). In the subintima of both early and advanced OA patients, there was not a statistically significant difference. These results were graphically summarized in Figure 2C.

**Figure 6:**
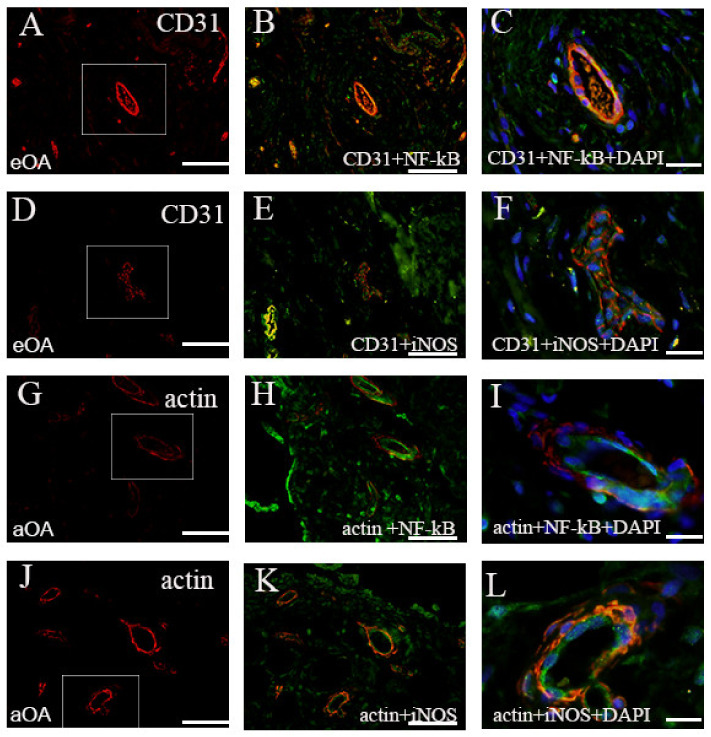
The synovial membrane of patients. CD31 positive cells (red) in the blood vessels (frame) (**A**). CD31+NF-kB+ (merge) in the endothelial cells (**B**). The merge of CD31+NF-kB+DAPI (blue) nuclear; stain (**C**). CD31 positive cells (red) in the blood vessels (frame) (**D**). CD31+iNOS+ (merge) in the endothelial cells (**E**). The merge of CD31+iNOS+DAPI (blue) nuclear stain (**F**). Actin positive cells (red) in the blood vessels (frame) (**G**). Actin+NF-kB+(merge) in the smooth muscle cells (**H**). The merge of Actin+NF-kB+DAPI (blue) nuclear stain (**I**). Actin positive cells (red) in the blood vessels (frame) (**J**). Actin+iNOS+(merge) in the smooth muscle cells (**K**). The merge of Actin+iNOS+DAPI (blue) nuclear stain (**L**). eOA—early OA; aOA—advanced OA. Magnification ×40 (first two columns), scale bar 50 µm and ×100 (last column), scale bar 20 µm.

The smooth muscle cells and endothelial cells the of blood vessel walls were only seen occasionally. NF-kB and iNOS were present in the endothelial and smooth muscle cells of the synovial blood vessels in early and advanced OA patients (Figure 6).

## 3. Discussion

Synovitis has been radiologically proven to be a prognostic factor for OA development [37,38], and innate immunity plays a paramount role in early OA advancement [28]. However, the pathogenic influence of synovial tissue inflammation on early and advanced OA is not clear [39,40]. Our previous study showed the higher grade of synovitis in patients with radiologically early OA compared to advanced eOA [11]. Additionally, in the same study, NF-kB and iNOS expression was observed to be greater in early OA compared to advanced OA. Now, as a continuation of the previous study, we wanted to elucidate the exact localization of these inflammatory factors while also examining the expression of MMP-9, being aware that MMPs are the most notable degradation enzymes in cartilage deterioration [24,26]. Our main findings indicate that macrophages are the most active cells in early OA, containing most of the NF-kB, which is responsible for the production of an abundance of proinflammatory factors. In advanced OA, leukocytes contain most of the NF-kB, becoming the leading proinflammatory cells. During early OA, iNOS is mostly located in macrophages, and in advanced OA, it is expressed in leukocytes, though it still maintains presence in macrophages.

In our study, nuclear NF-kB expression in early OA was mostly pronounced in the macrophages of the intima with moderate expression in the fibroblasts. This finding is consistent with finding of others confirming that hyperplasia of the intima of the synovial membrane is due to a highly increased number of fibroblasts and macrophages, with the latter being the main source of proinflammatory cytokines [27,29]. These cytokines, especially TNFa and IL-1, are mostly produced through the NF-kB transcription factor [19], and therefore, knowing the exact location of NF-kB, we can say which cells contribute the most to the inflammation present in the synovium during early OA. We found a high expression of NF-kB in the synovial macrophages, which is direct evidence for the involvement of macrophages in the pathogenesis of knee OA. Clearly, the NF-kB pathway is not the only one through which inflammatory cytokines can be produced, but it constitutes a major ratio [18,29]. Additionally, a considerable proportion of NF-kB is expressed by the fibroblasts. Fibroblasts are the most present cells in both normal and inflamed synovium [27]. Studies have shown that by depleting macrophages, fibroblast cytokine production was also downregulated due to cytokine cross-talk [29]. Our results show this pattern regarding the NF-kB expression in these two cell types. In advanced OA, NF-kB expression in macrophages dropped ten times, lowering the expression of inflammatory cytokines, while in fibroblasts, the decrease was significant but smaller. In the synovial subintima (stroma) during advanced OA, NF-kB was mostly expressed in the leukocytes, particularly in the lymphocytes, which is a novel finding. This might implicate that subintimal leukocytes express NF-kB during the late stages of OA, keeping the inflammatory process active through this stage. In advanced osteoarthritis, the systemic pattern of disease behavior is more notable. Perivascular, nodular lymphocyte infiltration appears in response to chronic inflammation, most likely due to the autoimmune component of the disease, which also partly confirms the systemic basis of the disease [41]. The expression of NF-kB in the smooth muscle cells and the blood vessel endothelium of the synovial tissue was negligible, which was expected. 

In immunohistochemical staining, iNOS is frequently used as a marker for activated macrophages, which signifies a consistent expression of this proinflammatory factor in macrophages while being active [28]. It is also secreted by fibroblasts, which constitute the dominant cellular component of the synovium [42]. iNOS expression in the intima of patients with early osteoarthritis followed a similar pattern as NF-kB, but not in in synovial the intima of advanced OA patients, where it was still expressed in the macrophages and leukocytes. This is in line with our previous study and other studies that show that the transcription of iNOS usually goes through the NF-kB pathway, but not entirely [11,19]. However, iNOS production in leukocytes in the synovial intima has grown exponentially in advanced OA. It is still unclear why, in advanced OA, most of the NF-kB presence was in the synovial subintima (stroma), whilst iNOS was mostly located in the synovial intima in the same stage of the disease.

In our study, we found MMP-9 to be predominantly produced by macrophages. Additionally, MMP production is strongly dependent on macrophage NF-kB activity, even when it is produced by fibroblasts [42,43]. Fibroblasts produce MMPs after being stimulated by cytokines, namely TNFa and IL-1 [44]. In line with these observations, in our study, we showed that in advanced OA, fibroblasts produce a higher percentage of total MMP-9 compared to early OA, but still less than the macrophages. A study by Amos et al. showed the decreased production of MMP-2, MMP-3, and MMP-9 once NF-kB had been blocked by IkB [18]. 

For this study, the synovium was taken from the medial and lateral gutters, parts of the knee joint with the most pronounced synovitis. The synovitis in patients with knee OA exhibits features of a T-cell immune response, with lymphocyte nodule numbers progressively growing proportionally with OA severity [41]. T-cells are mostly found in the synovial membrane and in the Hoffa fat pad of OA knees as proinflammatory cells [45]. These T-cells become dysfunctional phenotypes, thus contributing to the pathogenesis of knee OA [46]. With the achieved results, we favor the idea that both peripheral blood and tissue-infiltrating CD8 T-cells play an important role in the ongoing process of knee osteoarthritis [47]. These results suggest that B-cells and granulocytes may also be involved in the pathogenesis of knee OA, but, due to the scarce number of B-lymphocytes, their role is much smaller. Parts of the knee synovium in immediate contact with degrading cartilage are the locations of most lymphocytes [48].

Regarding the limitations of the study, the definition of the severity of knee OA was based solely on radiographs. The evidence so far does not show a strong correlation between radiographic changes and pain [49,50]. Some patients in the early OA group had bucket-handle meniscal tears with knee locking, which may not genuinely reflect synovitis due to OA. Even in the absence of OA, synovitis is also a feature of meniscal tears [51,52].

In conclusion, our study results provide information about different inflammatory cell localization in the synovial membrane of early and advanced OA patients compared to healthy individuals. The exact localization of the cells and stage of the disease when cells are active have an impact on the possible treatment of OA in its early stages. A direct association between radiological parameters and histologically proven tissue inflammation associated with different inflammatory cell immunophenotype will further elucidate OA pathophysiology.

## 4. Materials and Methods

### 4.1. Patients

The Ethics Committee of the School of Medicine, University of Mostar and the University Hospital Mostar, Bosnia and Herzegovina approved the study, and all patients gave written, informed consent. This study included 30 patients admitted to the Department of Orthopaedics and Traumatology of University Hospital Mostar. The inclusion and exclusion criteria were in accordance with the American College of Rheumatology Diagnostic and Therapeutic Criteria for knee OA [53], explained in detail in our previous study [11]. Four weeks prior to surgery, patients did not receive any anti-inflammatory drugs [54]. From the patients’ histories, we collected data about gender, age, body mass index (BMI), range of motion (ROM), knee axis, symptom duration in months (pain and contracture), and clinical stage of the disease by Western Ontario and McMaster Universities Arthritis Index (WOMAC) [55].

Twenty patients (over 40 years old) had primary OA, and they were divided into two groups according to radiological, Kellgren–Lawrence (K–L), classification [56]: ten patients with early OA and 10 patients with advanced OA. Early OA was considered as stage 1 and stage 2 according to K–L classification, while advance OA was stage 3 and stage 4 according to K–L classification. In the control group, there were ten younger aged patients (16–40 years) admitted for fresh meniscal injury without arthroscopically visible hyaline cartilage damage (grade 0 or 1), by the International Cartilage Repair Society classification (ICRS) [57]. In early radiographic OA, patients mostly complained about pain and sometimes knee locking. Here, we performed arthroscopies with the debridement of the deteriorated cartilage and abundant synovial membrane. When there was knee locking, we usually found medial meniscus bucket-handle tears, and a partial meniscectomy was performed. Arthroscopy, a minimally invasive procedure relieves some of the pain and prevents future knee locking in patients. In advanced radiographic OA, total knee endoprosthesis was implanted. 

### 4.2. Synovial Tissue Collection

A larger sample of synovial biopsies were taken during total knee arthroplasty from ten patients with advanced OA (stage 3 and stage 4 according to K–L classification). In all cases, standard medial parapatellar exposure was used. Synovial biopsies in other groups were performed arthroscopically. Samples were taken from ten patients with early OA and from the control group. All patients from the early OA group had their samples taken from the suprapatellar pouch, medial, and lateral gutters by direct visualization of the inflamed and hypertrophied tissue. Samples from control group were taken randomly from the same joint locations. Standard knee arthroscopic portals were used for the arthroscopy. 

### 4.3. Tissue Processing and Analysis

Tissue samples of the synovial biopsies were formalin-fixed, paraffin-embedded, serially sectioned (4 µm), and mounted on glass slides. Every 10th section underwent haematoxylin and eosin staining and were analyzed using an Olympus CX41 light microscope (Olympus, Tokyo, Japan). For every patient, there were 3 biopsies that were sectioned into ten tissue sections, making a total of 30 sections per patient. Three investigators analyzed the images of the synovial tissues independently.

### 4.4. Immunofluorescence

The remaining sections were deparaffinized in xylol and rehydrated in ethanol and distilled water, followed by cooking in a sodium citrate buffer (pH 6.0) for 10 min at 95 °C. Before the primary antibody application, non-specific staining was prevented by using Protein Block (ab64226; Abcam, UK). The appropriate combination of primary antibodies was incubated overnight in a humidified chamber (Table 1). The next day, the sections were rinsed in PBS and incubated in appropriate combinations of the secondary antibodies (Table 2) for one hour. After a final rinse in PBS, nuclei were stained using 4,6-diamidino-2-phenylindole (DAPI), and sections were then cover-slipped (Immuno-Mount, Thermo Shandon, Pittsburgh, PA, USA) and examined by a fluorescence microscope (Olympus BX51, Tokyo, Japan) equipped with a DP71 digital camera (Olympus, Tokyo, Japan). Images were taken at ×40 magnification and assembled using Adobe Photoshop. 

Double immunofluorescence with primary antibodies to NF-kB, iNOS, and MMP9 (Santacruz Biotechnology, Santa Cruz, CA, USA, SC-109 and SC-651, respectively) was used in combination with different specific cell type markers (Table 1) to determine the number of positive cells in the surface layer of cells (intima) and the underlying tissue (subintima) of the synovial membrane. For negative control, primary antibodies were excluded from the staining procedures. As a positive control, we used the lymph node and tonsil tissue. We only counted cells that displayed both markers in the same cell (red or green signal) in the nucleus or cytoplasm. The cell count was performed using the Olympus CellB (Olympus, Tokyo, Japan) and ImageJ software [58]. The total number of vimentin+/NF-kB+, CD68+/NF-kB+, LCA+/NF-kB+, vimentin+/iNOS+, CD68+/iNOS+, LCA+/iNOS+ and vimentin/MMP-9, CD68+/MMP-9, and CD5+/MMP-9 positive cells were calculated as number of cells per mm2 in the intima and subintima of the synovial membrane. The final total number per patient was the mean of 30 sections that were counted.

### 4.5. Statistical Analysis

The data were analyzed using SPSS17 software (SPSS Inc., Chicago, IL, USA). Nonparametric variables were presented as frequencies and percentages. Parametric variables were shown as median and interquartile range due to deviations from the normal distribution. To test differences between groups for categorical variables, we used chi-square test and Fisher’s exact test. To test differences between the parametric variables, we used the Mann–Whitney U test and Kruskal–Wallis test when the distribution of the data significantly deviated from normal. To test the correlation between the studied variables, Spearman’s correlation coefficient was used. Probability level *p* < 0.05 in all tests was taken as statistically significant.

## Figures and Tables

**Table 1:** Clinical characteristics of the examined groups.

	Control	Early OA	Advanced OA	*p* Value
Age (mean ± SD, years)	20.67 ± 2.69	58 ± 6.53	70.7 ± 3.30	
BMI (mean ± SD)	-	29.05 ± 4.68	29.74 ± 4.08	0.729
ROM (mean ± SD, degrees)	-	111.50 ± 21.86	93 ± 13.98	0.037 *
Duration (mean ± SD, years)	-	3.25 ± 2.80	14.5 ± 7.62	0.000 *
WOMAC (mean ± SD)	-	43.1 ± 21.64	61.3 ± 11.18	0.030 *

chi-square test, * *p* ˂ 0.05.

**Table 2:** Primary and secondary antibodies used.

Antibodies	Host	Dilution	Structures Identified by Antibodies	Source
SC-109 (polyclonal antibody)	Rabbit	1:200	Nf-ĸB p65	Santacruz Biotechnology (Santa Cruz, CA, USA)
SC-651 (monoclonal antibody)	Rabbit	1:200	iNOS	Santacruz Biotechnology (Santa Cruz, CA, USA)
A0150 (polyclonal antibody)	Rabbit	1:100	MMP-9	DAKO (Gloustrup, Denmark)
M0823 (monoclonal antibody)	Mouse	1:20	CD31 (endothelial cells of blood vessels)	DAKO (Gloustrup, Denmark)
M0851 (monoclonal antibody)	Mouse	1:40	Actin (smooth muscle cells of blood vessels)	DAKO (Gloustrup, Denmark)
M0725 (monoclonal antibody)	Mouse	1:50	Vimentin (fibroblasts)	DAKO (Gloustrup, Denmark)
M0876 (monoclonal antibody)	Mouse	1:75	CD68 (macrophages)	DAKO (Gloustrup, Denmark)
M0742 (monoclonal antibody)	Mouse	1:100	LCA (leukocytes)	DAKO (Gloustrup, Denmark)
M7194 (monoclonal antibody)	Mouse	1:50	CD5 (lymphocytes)	DAKO (Gloustrup, Denmark)
Rhodamine Goat AP124R	Mouse	1:100	Secondary antibody	MerckMillipore (Billerica, MA, USA)
Fluorescein Goat AP132F	Rabbit	1:100	Secondary antibody	MerckMillipore (Billerica, MA, USA)

## Data Availability

The study did not report any data.
